# Editorial: Case reports in heart surgery 2024

**DOI:** 10.3389/fcvm.2025.1692834

**Published:** 2025-10-29

**Authors:** Giuseppe Gatti

**Affiliations:** Cardiac Surgery Unit, Cardio-Thoracic and Vascular Department, Azienda Sanitaria Universitaria Giuliano-Isontina (ASUGI), Trieste, Italy

**Keywords:** left ventricular aneurysm, left ventricular thrombosis, mediastinitis, multidisciplinary approaches, multimodal imaging, case report, heart surgery

**Editorial on the Research Topic**
Case reports in heart surgery: 2024

For the second consecutive year, I had the pleasure and honor of coordinating the (fourth) Research Topic of “*Case Reports in Heart Surgery*” by Frontiers in Cardiovascular Medicine and Frontiers in Surgery. I am sincerely grateful to the Editors-in-Chief for their renewed trust.

As with the previous edition, the objective for the 2024 Research Topic was to feature unique cases of patients that present with an unexpected diagnosis, treatment outcomes, or clinical courses. Only original Case Reports that will significantly advance the field of cardiac surgery were considered, in my opinion and that of the Reviewers who collaborated with me throughout the manuscript review process. Rare cases with typical features, frequent cases with atypical features, and cases with a convincing response to new treatments were included in the present Research Topic.

The (undeclared) goal was also to match the excellent results achieved by the 2023 edition of the Research Topic, which has received over 26,000 views to date. I am confident! The contributions selected in this 2024 edition are also rare and original clinical cases that could offer readers of Frontiers many points of interest and reflection.

The Research Topic consists of 15 articles written by a total of 80 Authors from seven countries (China, Colombia, Russia, Singapore, Switzerland, Türkiye, and the United States) spanning four different continents. To date, the Research Topic has already received over 17,000 views.

The main issues addressed in the present Research Topic are

- The growing importance of minimally invasive surgery and interventional techniques and technologies (Tian et al., Peng et al., Idu et al., Çetinarslan and Saba, Bidovecet al., Xia et al., Aigumov et al., Chen et al.), and, inevitably, of their complications (Tian et al., Peng et al., Idu et al.). Obviously, the “minimally invasive” concept should be understood in a broad sense, including minimal, mechanical, and/or biological (humoral and cellular) invasiveness;
- The essential need for multimodal imaging for complex cardiovascular lesions (Tian et al., Lianget al., Shan et al., Peng et al., Idu et al., Bidovecet al., Xia et al., Yang et al., Aigumov et al., Chen et al., Ma et al.);
- The essential need for a multidisciplinary approach to diseases involving multiple organs or systems, including the heart (Wang et al., Lianget al., Peng et al., Nunez-Ordonez et al., Idu et al., Çetinarslan and Saba, Bidovecet al., Xia et al., Rodgers et al., Yang et al., Chen et al., Ma et al.). To improve patient outcomes, in addition to the cardiologist and cardiac surgeon, other healthcare professionals, each within their own area of expertise, should also be involved in the management of complex cardiac patients and/or diseases. This is in accordance with the Latin maxim, “Unicuique suum” (To each his/her own);
- The significant role of sometimes neglected (albeit life-threatening) complications such as sternal wound infections (Wang et al., Idu et al., Rodgers et al.) and pericardial effusion (Idu et al.);

An interesting report on two unresolved cases of late aortic endograft infection complicated by peri-aortic abscess (Peng et al.) was (perhaps improperly) included in the present Research Topic of case reports on heart surgery to familiarize cardiac surgeons with endovascular repair of the thoracic aorta and its rare but catastrophic complications involving the mediastinum.

I synthesized the main message of each contribution to the present Research Topic in Table 1.

**Table 1:** Case reports in heart surgery 2024^a^.

Paper number	First author	Title	Publication date	DOI	Total views^b^	Main findings	Keywords
1.	Yunfei Tian	Case Report: Minimally invasive management of suspected active bleeding from intercostal vessel after axillary thoracotomy ventricular septal defect repair: an application of the Foley catheter	May 27, 2025	doi.org/10.3389/fcvm.2025.1511221	640	Injury to the intercostal vessels during an axillary thoracotomy can be a serious complication of minimally invasive cardiac surgery. The Authors reported a case of major bleeding following right axillary thoracotomy that was successfully treated using a Foley catheter inserted through the chest wall.	Axillary thoracotomy; Foley catheter; Minimally invasive surgery
2.	Zhaohui Wang	Case Report: Deep sternal wound infection caused by *Mycoplasma hominis* after cardiac surgery	May 12, 2025	doi.org/10.3389/fcvm.2025.1538389	759	The Authors reported a case of DSWI after cardiac surgery caused by *Mycoplasma hominis*.	DSWI; *Mycoplasma hominis*
3.	Chunshui Liang	Successful treatment of massive biventricular thrombi associated with myocarditis: a case report	Apr 28, 2025	doi.org/10.3389/fcvm.2025.1530548	797	In a young man with a stroke, massive bi-ventricular thrombosis associated with myocarditis was successfully treated with drugs alone, according to a multidisciplinary approach.	Massive bi-ventricular thrombosis; Multidisciplinary approach; Myocarditis
4.	Jianggui Shan	Case Report: “Dumbbell” giant right coronary artery ectasia with right atrial fistula	Feb 28, 2025	doi.org/10.3389/fcvm.2025.1498359	943	Type IV giant ectasia of the right coronary artery, right atrial fistula, epicardial muscle bridge, and functional mitral regurgitation were all successfully repaired in a middle-aged woman. The Authors discussed the origin and developing stages of this complex cardiac lesion.	Cardiac fistula; Giant coronary aneurysm
5.	Danping Peng	Aortic endograft infection after thoracic endovascular aortic repair: two case reports and literature review	Feb 25, 2025	doi.org/10.3389/fcvm.2025.1549613	919	Two unresolved cases of late aortic endograft infection complicated by peri-aortic abscess involving the posterior mediastinum were presented by the Authors, who emphasized the role of strict follow-up after TEVAR to prevent late life-threatening complications. A literature review of the topic was also reported.	Mediastinitis; Peri-aortic abscess; TEVAR
6.	Nicolas Nunez-Ordonez	Case Report: A usual procedure in an unusual situation: a patient with a rare Ehlers-Danlos/ osteogenesis imperfecta overlap undergoing aortic valve replacement	Feb 24, 2025	doi.org/10.3389/fcvm.2025.1480363	1,071	A case of Ehlers-Danlos/Osteogenesis Imperfecta Overlap Syndrome undergoing aortic valve replacement for aortic regurgitation was presented. The Authors emphasized the importance of a multidisciplinary approach to managing patients with rare genetic disorders.	Ehlers-Danlos syndrome; Multidisciplinary approach; Osteogenesis imperfecta; Overlap syndrome
7.	Muhammad Idu	Case Report: Late presentation of post-coronary artery bypass surgery pericardial effusion heralding mediastinitis with tracheocutaneous fistula	Feb 13, 2025	doi.org/10.3389/fcvm.2025.1483395	1,023	A case of late post-CABG pericardial effusion, complicated by mediastinitis and tracheocutaneous fistula, was illustrated.	Mediastinitis; Pericardial effusion; Tracheocutaneous fistula
8.	Özge Çetinarslan	Case Report: Staged surgical management in ESRD: off-pump CABG followed by renal transplantation to enhance graft survival	Feb 7, 2025	doi.org/10.3389/fcvm.2025.1486771	798	Two patients scheduled for renal transplantation because of end-stage renal failure underwent uneventful off-pump CABG without blood transfusion. The Authors emphasized the role of off-pump surgery, compared to on-pump surgery, in minimizing blood transfusions, which could eventually increase the risk of graft rejection.	Blood transfusion; Graft rejection; Off-pump CABG; Minimally invasive surgery
9.	Jan Bidovec	Case Report: PCI of the left coronary artery after salvage operation in a comatose patient with an acute type A aortic dissection	Feb 6, 2025	doi.org/10.3389/fcvm.2025.1516152	1,128	A two-stage, hybrid treatment involving ascending aorta plus aortic arch replacement, along with intravascular ultrasound-guided stenting of the left main, was performed on a hemiplegic elderly woman with bilateral carotid artery and right vertebral artery occlusion owing to acute aortic dissection. Since there was no residual neurological deficit, the Authors highlighted the importance of a multidisciplinary approach.	Acute aortic dissection; Hybrid treatment; Multidisciplinary approach; Neurological deficit
10.	Jianming Xia	Case Report: Surgery combined with extracorporeal membrane oxygenation for acute type A aortic dissection complicated with acute myocardial infarction	Jan 31, 2025	doi.org/10.3389/fcvm.2025.1463764	865	ECMO was successfully used as a perioperative hemodynamic support measure for three patients with acute aortic dissection complicated by right ventricular myocardial infarction.	Acute aortic dissection; ECMO; Peri-operative management
11.	Jeffrey Rodgers	Case Report: *Fusarium falciforme* pericardial and sternal wound infection following orthotopic heart transplantation	Jan 6, 2025	doi.org/10.3389/fcvm.2024.1480392	768	The Authors reported on the successful treatment of *Fusarium falciforme* mediastinitis in a heart transplant recipient.	*Fusarium falciforme;* Heart transplantation; Mediastinitis
12.	Yang Yuehang	Aortic valve replacement in a bicuspid aortic valve patient followed by reoperation for ascending aorta rupture: a case report	Dec 11, 2024	doi.org/10.3389/fcvm.2024.1471686	889	One case of ascending aortic rupture and aortic-right atrial fistula occurred after a replacement of stenotic BAV combined with ascending aortoplasty for mild (43 mm) aortic dilation. Replacement of the ascending aorta was finally performed. Given the increased risk of severe future complications, the Authors raised awareness among the readers about the need to consider a strategy for resolving ascending aortic replacement in the presence of diseased BAV, even with aortic dilation cutoffs lower than 45 mm (as recommended by the most recent guidelines recommendations).	Ascending aortic replacement; Ascending aortic rupture; Ascending aortoplasty; BAV; Guidelines
13.	RN Aigumov	Geometric reconstruction of the left ventricle on a beating heart through a minimally invasive approach from the left anterolateral thoracotomy: case report	Dec 4, 2024	doi.org/10.3389/fcvm.2024.1507222	1,043	A post-infarct aneurysm of the LV, complicated by endocavitary thrombosis, was successfully repaired through left anterolateral thoracotomy using the “beating heart” technique. According to the Authors, this minimally invasive approach is safe and could improve LV geometric reconstruction.	Beating heart surgery; LV aneurysm; Minimally invasive surgery
14	Yu Chen	Case Report: 3D imaging-assisted minimally invasive hybrid closure surgery of a complex coronary artery fistula	Nov 22, 2024	doi.org/10.3389/fcvm.2024.1439263	803	A pediatric case of successful, single-stage hybrid closure of a complex coronary artery fistula was reported. The Authors emphasized the decisive role of preoperative three-dimensional imaging and intraoperative transesophageal echocardiography.	Complex coronary artery fistula; Hybrid treatment; Minimally invasive surgery; Three-dimensional imaging
15	Hongjia Ma	Case Report: Surgical management of traumatic giant coronary artery pseudoaneurysm with pericardial patch repair and ostium isolation	Nov 8, 2024	doi.org/10.3389/fcvm.2024.1462557	633	A complex surgical repair of a giant coronary artery pseudoaneurysm caused by trauma was reported. The correct anatomical reconstruction was confirmed with transthoracic echocardiography at one month after surgery.	Cardiac trauma; Giant coronary pseudoaneurysm

BAV, bicuspid aortic valve; CABG, coronary artery bypass grafting; DOI, digital object identifier; DSWI, deep sternal wound infection; ECMO, extracorporeal membrane oxygenator; LV, left ventricle/ventricular; TEVAR, thoracic endovascular aortic repair.

^a^ Case reports are listed in order of publication date.

^b^ 22 Aug, 2025.

In the 2023 Editorial (1), I stated: “Personally, I am particularly fond of the Case Reports sections of surgical Journals because they often include interesting and innovative contributions. The clinical presentation, diagnostic process and effective surgical treatment of rare conditions offer the reader stimulating food for thought. Sometimes there are reported cases of failure but of great educational value. However, Case Reports sections are increasingly rare nowadays in scientific Journals where more value is placed on large-scale studies such as multicenter studies, randomized controlled trials or meta-analyses. (..) Both for Heart Surgery and Interventional Cardiology, the most advanced frontiers of the disciplines are often glimpsed by analyzing Case Reports!”. [SIC] Today, after reviewing dozens of Case Reports for Frontiers, I agree with myself more than ever.

In closing, I would like to sincerely thank all the valuable Reviewers and Co-editors who helped me with my task. I have certainly learned a great deal from them throughout this experience. In addition, I would like to thank all the members of the Editorial Offices of the two valuable scientific Journals for supporting me at every step of this experience. Thank you again to everyone!
